# Estimation of Task-Related Dynamic Brain Connectivity via Data Inflation and Classification Model Explainability

**DOI:** 10.1007/s12021-025-09733-6

**Published:** 2025-06-03

**Authors:** Peter Rogelj

**Affiliations:** Faculty of Mathematics, Natural Sciences and Information Technologies, University of Primorska, Glagoljaška 8, Koper, SI-6000 Slovenia

**Keywords:** EEG, Functional connectivity, Data inflation, Classification, Explainability, Saliency maps

## Abstract

Study of brain function often involves analyzing task-related switching between intrinsic brain networks, which connect various brain regions. Functional brain connectivity analysis methods aim to estimate these networks but are limited by the statistical constraints of windowing functions, which reduce temporal resolution and hinder explainability of highly dynamic processes. In this work, we propose a novel approach to functional connectivity analysis through the explainability of EEG classification. Unlike conventional methods that condense raw data into extracted features, our approach inflates raw EEG data by decomposition into meaningful components that explain processes in the application domain. To uncover the brain connectivity that affects classification decisions, we introduce a new method of dynamic influence data inflation (DIDI), which extracts signals representing interactions between electrode regions. These inflated data are then classified using an end-to-end neural network classifier architecture designed for raw EEG signals. Saliency map estimation from trained classifiers reveals the connectivity dynamics affecting classification decisions, which can be visualized as dynamic connectivity support maps for improved interpretability. The methodology is demonstrated on two publicly available datasets: one for imagined motor movement classification and the other for emotion classification. The results highlight the dual benefits of our approach: in addition to providing interpretable insights into connectivity dynamics it increases classification accuracy.

**Supplementary Information:**

The online version contains supplementary material available at 10.1007/s12021-025-09733-6.

## Introduction

Electroencephalography (EEG) is a noninvasive method for monitoring brain activity, offering valuable insights across a broad range of applications, including medical diagnostics, the study of brain function, the effects of environmental factors on individuals, and even controlling devices through brain-computer interfaces. However, EEG signals are highly complex, and only a fraction of their information can be understood without the use of signal processing and computerized analysis. The improvements in EEG processing methods have two primary objectives: first, to enhance the ability to automatically recognize and classify data based on mental states or events, and second, to uncover what is happening inside the brain that is linked to these states and events.

There are two primary approaches to EEG processing: the conventional approach, which relies on manual extraction of hand-crafted features, and the modern approach, which leverages deep learning (Saeidi et al., 2021; Aggarwal and Chugh, 2022). Hand-crafted features are typically designed based on our knowledge of brain function and include techniques like power spectrum analysis, time-frequency analysis, waveform feature detection, and connectivity analysis. These models offer the advantage of being interpretable, allowing researchers to link classification outcomes back to specific brain properties, thereby deepening our understanding of brain function. In contrast, deep learning methods (Roy et al., 2019; Craik et al., 2019; Gemein et al., 2020; Köllőd et al., 2023; Ellis et al., 2024; Yang and Liu, 2024; Zhao et al., 2024; Lawhern et al., 2018) tend to achieve higher classification accuracy but lack transparency, making the reasoning behind their decisions more difficult to explain.

In recent years, there has been a growing emphasis on the explainability of deep learning models, a critical factor for advancing neuroscientific research and enhancing our understanding of cognitive processes. For a comprehensive review and comparison of various approaches applicable to EEG processing, refer to Sujatha Ravindran and Contreras-Vidal (2023). Some researchers advocate for the use of black-box interpretability methods like LIME (Hussain et al., 2023), while others focus on analyzing convolutional kernels to uncover the learned network patterns in the frequency domain (Salami et al., 2022). Another promising direction involves explaining deep models’ internal states through human-aligned concepts, using techniques such as Concept Activation Vectors (CAVs) (Gjølbye et al., 2024). However, the most commonly employed approach is analyzing the importance of input data for classification decisions using saliency maps (Farahat et al., 2019; Ieracitano et al., 2022; Wang et al., 2022; Mortier et al., 2023). This technique is often applied to highlight the relevance of raw signal samples in the time domain, though it can also be used on pre-extracted features like frequency components (Wang et al., 2024; Lopes et al., 2023) or connectivity matrices.

The choice of approach often depends on our current understanding of the principles underlying brain function. One key concept is the task-related switching between intrinsic brain networks (Lynn and Bassett, 2019; Shaw et al., 2021). The brain can be viewed as a highly interactive complex network, which can be studied by detecting the synchronization of neural activity across different regions (Hövel et al., 2020). This synchronization is often measured using various functional connectivity estimation methods, with some of the most promising techniques being phase locking value (PLV) (Lachaux et al., 1999), weighted phase locking index (wPLI) (Vinck et al., 2011), complex Pearson correlation coefficient (CPCC) (Šverko et al., 2022a) and Granger causality (GC) (Friston et al., 2013). For a more in-depth review of connectivity methods, see Chiarion et al. (2023). The majority of these methods are undirected, despite the neuronal influence being understood to be predominantly directed. Additionally, they all face challenges in capturing the dynamic nature of brain connectivity with high temporal precision (Šverko et al., 2022b). These methods employ a windowing function to statistically analyze the electrode relations. Using narrow windows results in high noise, while wider windows reduce temporal resolution. For analysis of resting state data, the typical window sizes range from 30 to 240 seconds (Hutchison et al., 2013; Fu et al., 2019) while for dynamic processes estimations the most common window size is one second (Phan et al., 2024; Panwar et al., 2021). This highly limits the dynamic capabilities of connectivity estimation methods and can be critical for brain network-state analysis (Ermolova et al., 2021). Finally, the additional significant challenge is determining which specific connections are associated with particular cognitive processes, which current methods do not address.

In this paper, we introduce a novel methodology inspired by the concept of intrinsic brain network switching, aimed at uncovering how brain regions dynamically interact to perform complex tasks. Unlike conventional dynamic connectivity estimation methods, which rely on temporal windowing and are limited in capturing rapid connectivity changes, our approach achieves a temporal resolution matching the EEG sampling rate. This enables a more precise analysis of brain dynamics while focusing on specific brain processes.

The paper is structured as follows. In Section Methods, we present our methodology, which involves three key steps:

- Data preparation using a newly proposed Dynamic Influence Data Inflation method (DIDI).
- Integration of a deep neural network classifier for accurate classification.
- Explanation of classification decisions to reveal task-related dynamic connectivity through saliency map computation.

These three steps collectively constitute our proposed approach, referred to as Dynamic Influence Data Inflation with Neural Network explainability (DIDI-NN). In Section Results, we demonstrate the proposed methodology on two EEG classification tasks:

- Imagined motor movement classification, showcasing dynamic analysis, and
- Emotion classification, focusing on static analysis.

In Section Discussion we highlight the strengths of the proposed approach, discuss its limitations, and outline opportunities for future enhancements. The paper concludes with a summary of our findings and key takeaways.

## Methods

Our proposed approach to reveal detailed dynamic connectivity related to specific brain processes is based on connectivity-grounded explanations of classification decisions. It can be presented as a three-step process, see Fig. 4. It starts with the preparation of the data, where raw EEG data get inflated by computing dynamic influence signals. The expanded data are then flattened into a 2D structure and, in the second step, fed into a deep neural network classifier. Class labels are not the only meaningful classification model output. In the additional step, the classification decision is explained by extracting a dynamic connectivity support map that shows which connections at which time most influence the classification decisions. This information can be further used to study brain processes from the neuroscientific perspective.

### Dynamic Influence Data Inflation

We define the dynamic influence data inflation method (DIDI), inspired by Granger causality (GC), a widely used directional connectivity measure. GC, when applied to EEG, estimates the improvement of prediction for a signal at one electrode $x_i$ by using the information of signal at the other electrode $x_j$, where *i* and *j* are electrode indexes for the influenced electrode and for the influencing electrode respectively. GC describes signal relations using vector autoregression (VAR) and based on available data builds a univariate model Eq. 1 and a bivariate model Eq. 2:

1 $$\begin{aligned} x_i(t) = \sum _{n=1}^{N} a(n) x_i(t-n) + \epsilon _i(t) \end{aligned}$$

2 $$\begin{aligned} {\begin{matrix} x_i(t) = & \sum _{n=1}^{N} a'(n) x_i(t-n) \\ +& \sum _{n=1}^{N} b(n) x_j(t-n) + \epsilon _{i,j}(t) \end{matrix}} \end{aligned}$$

Here, *N* is the model order, *a*, $a'$ and *b* are autoregression model coefficients, while $\epsilon _1$ and $\epsilon _{1,2}$ are univariate and bivariate autoregression errors respectively. In the process of modeling, the errors are minimized for a certain time window, or for the overall signal pair in the case of time-invariant modeling. Afterward, GC can be estimated as:

3 $$\begin{aligned} GC(i,j) = \log \left( \frac{var(\epsilon _i)}{var(\epsilon _{i,j})} \right) \end{aligned}$$

The errors represent the deviation from the system’s natural behavior and are often referred to as innovation process signals. Note that knowing the model parameters, the actual signal $x_i(t)$ can be fully reconstructed from the innovation process signals $\epsilon _i(t)$ or $\epsilon _{i,j}(t)$. In this work, we consider the models to be time-invariant (static) and all the dynamics can be explained by the innovation processes. This implies that the brain state is reflected in model parameters and activities in the innovation processes.

Following the approach of GC, bivariate model parameters $a'$ may differ from univariate parameters *a* whenever some component of signal $x_i$ is in any way related to $x_j$. This makes the bivariate model difficult to interpret. To overcome this problem, in this work, we preserve the univariate parameters when building the bivariate model ($a'=a$). Considering system linearity presumed by the VAR models, using the univariate parameters for bivariate regression does not affect the estimated innovation signal $\epsilon _{i,j}$. However, the obtained bivariate parameters *b* then get some wider meaning; they represent a regression model for predicting the univariate innovation process $\epsilon _{i}$ by the influencing signal $x_j$:

4 $$\begin{aligned} \epsilon _i(t) = \sum _{n=1}^{N} b(n) x_j(t-n) + \epsilon _{i,j}(t) \end{aligned}$$

As such, the innovation signal $\epsilon _{i,j}(t)$ represents a part of the overall univariate innovation signal $\epsilon _{i}(t)$ that cannot be explained by the influence of signal $x_j$. This also means that we can now estimate the directed influence signal $c_{i,j}$ as the contribution of signal $x_j$ to the univariate innovation signal of $x_i$:

5 $$\begin{aligned} c_{i,j}(t) = \epsilon _{i}(t) - \epsilon _{i,j}(t). \end{aligned}$$

**Fig. 1:**
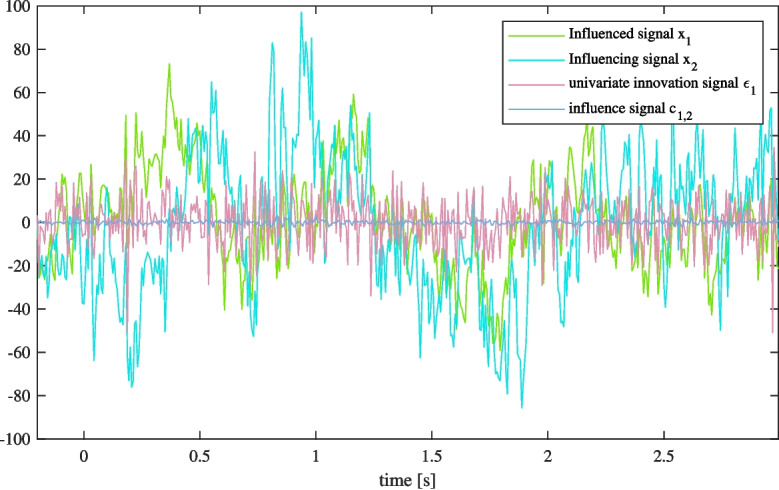
An example of univariate innovation signal $\epsilon _1$ (pink) and influence signal $c_{1,2}$ (blue) extracted from influenced signal $x_1$ (green) and influencing signal $x_2$ (cyan) for a single epoch of an imaginary movement classification experiment

When analyzing EEG data we compute influence signals for every pair of two electrodes. They are expected to explain most of the brain dynamics except for the processes unrelated to connectivity between the electrodes, which can be triggered by brain regions that are not monitored by EEG, or by external stimuli. Whenever a complete description of EEG dynamics is needed, e.g. when the goal is to gain high classification accuracy, we have to combine influence signals with signals that include the remaining electrode innovations. In this work we supplement them with univariate innovation signals $\epsilon _i(t)$. With this, the same connectivity information is actually presented twice, i.e. extracted in influence signals and unextracted in the added supplemental innovation signals. However, for further study of dynamic connectivity, connectivity-related information must be included in the extracted form, i.e., as influence signals. An example of innovation and influence signals extracted for a single epoch is shown in Fig. 1.

**Fig. 2:**
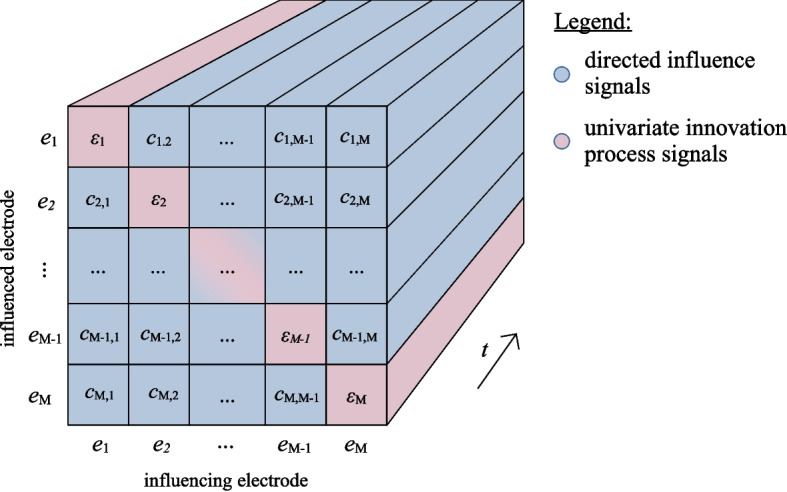
Inflated EEG data as a 3D array with dimensions corresponding to influenced electrodes, influencing electrodes and samples in time. The data includes the directed influence signals $c_{i,j}$ shown in blue and the univariate innovation process signals $\epsilon _i$ in pink

We collect all the previously described signals into a 3-dimensional array, i.e. inflated EEG data, where the dimensions correspond to the index of influenced electrode *i*, the index influencing electrode *j* and the sample number in time *t*, see the illustration in Fig. 2.

The autoregressive model order *N* is commonly selected by minimizing information criteria such as the Akaike Information Criterion (AIC) (Akaike, 1974). However, while such criteria are effective for reducing univariate prediction errors, they may not optimally capture the bivariate dynamics essential for functional connectivity estimation. In practice, the choice of model order must balance model accuracy, computational stability, and the temporal resolution required to capture relevant neural dynamics. Previous studies suggest that higher orders are preferable in modeling nonlinear systems (Winterhalder et al., 2005) and accounting for lags due to neuronal delays (Seth et al., 2015). As long as the number of samples is sufficient (typically at least 10 times the model order), using a higher order does not negatively impact stability and may improve the estimation of directed interactions. A widely accepted heuristic is to choose an order that corresponds to a temporal window of approximately 100–200 ms, which we follow in this study.

Let us summarize the implementation steps for dynamic influence data inflation (DIDI):

1. Define the order of the regressive models *N*. For EEG analysis, the order typically corresponds to a time window of approximately $100~-~200~ms$ as a compromise between model accuracy and computational efficiency. 
2. Compute the univariate autoregressive model parametereters *a*(*n*) indpendently for each EEG electrode signal $x_i(t)$ and compute the univariate innovation signals $\epsilon _{i}(t)$ as autregressive errors, see Eq. 1.
3. For each electrode pair of influenced electrode *i* and influencing electrode *j* compute:
  the regression model *b*(*n*) for predicting innovation signal $\epsilon _{i}(t)$ with electrode signal $x_i(t)$ as defined in Eq. 4,
  the regression error $\epsilon _{i,j}(t) \!=\! \epsilon _i(t) \!-\! \sum _{n=1}^{N}\! b(n) x_j(t\!-n)$,
  the directed influence signal $c_{i,j}(t)$ using Eq. 5.
4. Collect the computed directed influence signals $c_{i,j}(t)$ and univariate innovation signals $\epsilon _{i}(t)$ into the inflated data structure, as shown in Fig. 2.

### Neural Network Classification

Our approach to classifying EEG data takes a fundamentally different direction from conventional methods. Rather than compacting raw data into features, we propose using inflated EEG data, where each raw signal is represented as a collection of multiple signal components. This approach allows us to derive meaningful explanations for classification decisions. Additionally, when the information content of these components is closely related to the underlying processes being classified, it has the potential to enhance the model’s classification performance.

In this work, we focus on brain processes where functional connectivity plays a critical role. The previously described influence signals are as components of innovation signals particularly relevant for both accurate classification and meaningful interpretation in such contexts.

Several different neural network architectures were proposed for the classification of raw EEG data. Different architectures may be better tailored for specific EEG classification tasks. In this work, we do not focus on the selection or comparison of neural network architectures, but rather on the approach to employing them to reveal the connectivity-grounded explanations for specific brain processes by explaining the classifiers’ decisions. Any end-to-end neural network classification solution designed for EEG data could be employed. For our experiments, we decided to use the EEGNet architecture (Lawhern et al., 2018), which has already proved its capabilities in many different classification tasks, and is one of the most recognized architectures for EEG data classification. There are several alternatives, e.g., ShallowConvNet or DeepConvNet (Schirrmeister et al., 2017).

**Fig. 3:**
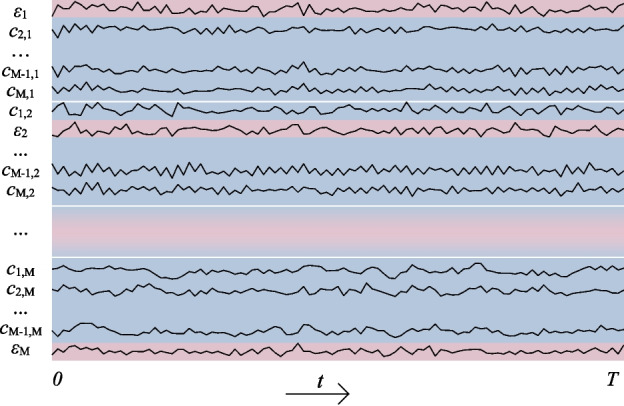
Input to the EEGNet neural network classifier is a 2D array, obtained by flattening the 3D inflated EEG data array (column-wise)

End-to-end EEG classifiers require input to be a 2D array of signals in time. Consequently, we need to flatten our 3D inflated EEG data (Fig. 2) into a 2D array by merging the first two dimensions (column-wise). Thus, the number of channels in EEGNet was set to the number of signal components in the inflated EEG data $M \times M$. The number of samples in time *T* was kept in the remaining dimension. For the Illustration of the flattened data see Fig. 3.

### Explanation of Classification Decisions By Dynamic Connectivity

The need to explain modern classification processes has led researchers to propose various approaches for analyzing neural network classifiers. One of the most widely used techniques is the computation of saliency maps, originally developed in the field of image classification to assess the spatial contribution of each part of an image to a particular classification decision. Different saliency map computation methods offer varying levels of detail and sharpness in estimating feature importance. In this work, we use the ’GradientExplanation’ method as implemented by Adebayo et al. (2018), which is designed to explain 2D images and is compatible with the EEGNet input data structure. In our approach, a saliency map represents the estimated significance of each inflated data component at every time sample in determining the classification outcome. The significance of influence signals highlights dynamic connectivity between corresponding electrode regions, while the significance of univariate innovation signals reflects mental processes occurring at individual electrode sites. When visualized on an electrode map, this representation is referred to as a dynamic connectivity support map. Since such maps can be generated for different time delays, they can also be presented as video sequences, providing a clearer and more intuitive depiction of brain dynamics. This information holds potential for advancing neuroscientific research by offering deeper insights into neural processes.

When analyzing responses to stimulation events (ERPs), the temporal reference provided by these events enables a more precise statistical analysis of brain connectivity response dynamics. In contrast, when examining longer-term or steady-state brain processes, the lack of a clear temporal reference restricts detailed time-resolved analysis. In such cases, the focus shifts to identifying general, time-invariant characteristics of brain activity. This can be achieved by averaging saliency maps over time, resulting in a single static connectivity support map that represents the overall connectivity patterns for an entire EEG data sequence.

**Fig. 4:**
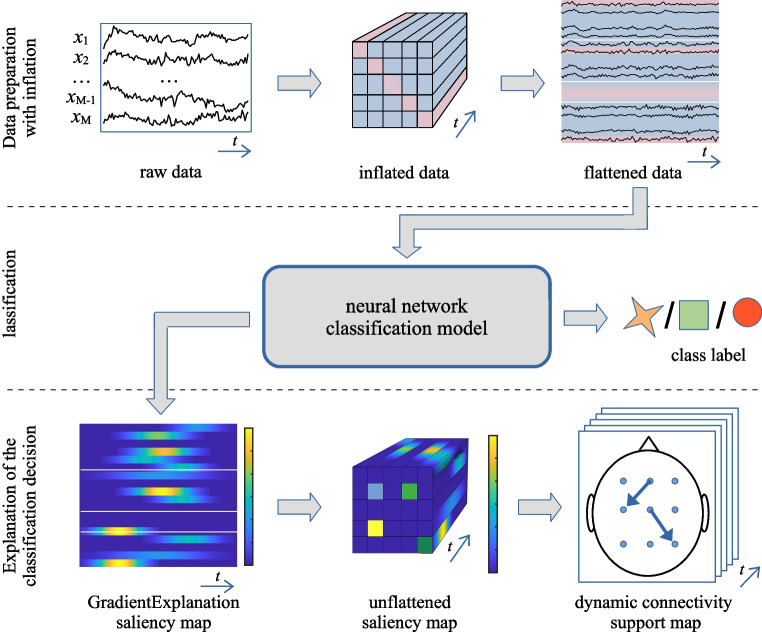
The task-related connectivity analysis process begins with dynamic influence data inflation (top), followed by training a classification model to classify the inflated input data (middle), and concludes with extracting gradient-based saliency maps, which can be visualized as dynamic connectivity support maps (bottom)

Both dynamic and static representations of task-related connectivity can be further explored through statistical analyses, such as averaging across multiple trials, specific subject groups, or an entire population. This approach (Fig. 4) provides a broader perspective on brain activity patterns and their relevance to the classification task, offering deeper insights into underlying cognitive processes.

## Results

In this section, we evaluate the impact of the proposed data preparation technique on classification accuracy and the ability to uncover task-related dynamic neural connectivity insights. Classification accuracy is a crucial metric, as it reflects the reliability of the extracted connectivity information.

We compared the accuracy of dynamic influence data inflation (DIDI) with that of raw EEG data (RAW) across four different classification tasks. Three of these tasks involved classifying motor movement activity in response to visual targets, while the fourth focused on classifying emotions elicited by carefully selected emotion-inducing film clips.

To demonstrate the ability of DIDI to reveal task-related connectivity, we analyzed one task from each category. A dynamic analysis was conducted on one of the event-based motor tasks, while the emotion classification task was used to illustrate static connectivity support analysis.

**Fig. 5:**
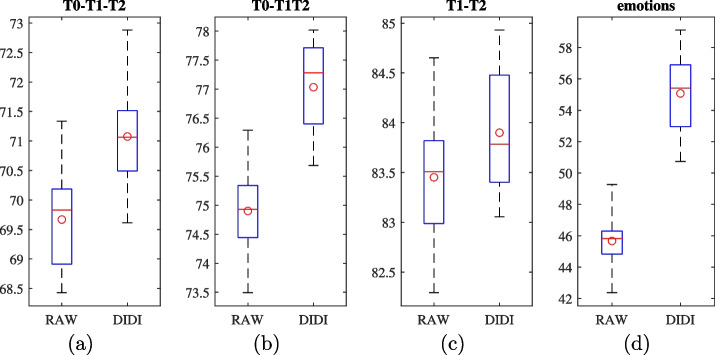
Accuracy distributions for three imaginary movement classification tasks (a, b and c) and emotion classification task (d), for both methods (RAW and DIDI inputs). Red circles indicate mean accuracies of each method

In all experiments, only 19 electrodes from the international 10-20 system were utilized, even though the original datasets included 64 or 62 electrodes in 10-10 system setups. The EEGNet neural network classifier was used in all cases, with its meta-parameters set according to the authors’ recommendations. No additional meta-parameter optimization was performed, except for adapting kernel sizes to the sampling rate as suggested. While this may have led to suboptimal classification results, it ensured a fair comparison between different data preparation methods by eliminating potential biases. For practical applications, optimizing neural network meta-parameters is highly recommended, and alternative neural network architectures may also be considered.

### Dynamic Analysis of Imagined Motor Movement EEG

Dynamic analysis is demonstrated in the classification of imagined motor movement. A subset of the freely available EEG Motor Movement/Imagery Dataset (Schalk et al., 2004, 2009) from PhysioNet (Goldberger et al., 2000) was used, selecting only the dataset’s task nr. 2 (imagine opening and closing left or right fist). All the provided data are recorded using a 64-channel EEG system, while for our experiment the number of electrodes was reduced to 19, keeping only electrodes of the International 10-20 system. The sampling rate was 160 Hz. The data has already been preprocessed by the authors of the dataset, so the only preprocessing step applied before defining the classification inputs with potential data inflation was average rereferencing. The order for the regression models was 20, which corresponds to a 125 *ms* time window of historical signal values. The data of 106 subjects were used. Each subject conducted several experiment runs, three of them for the selected task of imagined movement (experiment runs 4, 8, and 12). Each run consists of interchanging T1 and T2 events of showing a trigger target on a screen that instructed the subject to start imagining opening and closing left or right fist, respectively. After 4 seconds the trigger target was removed from the screen, which is recorded as event T0, and the subject stopped the imagined motor movement. The rest in between the imagined motor movements also lasted 4 seconds.

The differentiation between the two triggered imagined fist movements and the triggered resting is analyzed in three classification tasks:

- **T0-T1-T2** classification of rest T0, imagined left fist movement T1 and imagined right fist movement T2. Note that there are twice as many T0 events than T1 and T2 events, so the dataset is not ballanced.
- **T0-T1T2** differentiation between rest (events T0) and imagined movement (events T1 and T2). The dataset in this experiment is balanced.
- **T1-T2** differentiation between left and right imagined fist movements, considering only events T1 and T2. The dataset in this experiment is balanced.

There were 90 events per subject (50% T0, 25% T1 and 25% T2). For each event, we extracted an event-related potential (ERP) in the duration from 0.2 *s* before to 3 *s* after the event. The dataset was randomly split in a subject invariant (intersubject) manner into train and test subsets, such that 74 subjects (70%) were used for training and 32 subjects (30%) for testing. The additional validation subset was not needed as the neural network’s meta-parameters were set according to EEGNet recommendations and were not being tuned. The same data split was used for all the experiments, classification tasks, and for both methods regarding the data inputs (RAW and DIDI). Due to the randomness in neural network training, each of the three classification tasks (T0-T1-T2, T0-T1T2, and T1-T2) was repeated, i.e., trained and evaluated, 20 times for each method. The obtained accuracies were statistically analyzed and are presented in Fig. 5 (a,b and c) and Table 1. Note that the dataset for task T0-T1-T2 was not balanced; always classifying to T0 would be 50% accurate. As such all three tasks have a zero-rate accuracy of 50%.

**Table 1:** Mean accuracies achieved using different methods for the three imagined movement classification tasks and emotion classification task

	T0-T1-T2	T0-T1T2	T1-T2	emotions
RAW	69.67	74.90	83.45	45.67
DIDI	71.08	77.03	83.90	55.07

The accuracy results across all runs, tasks, and both methods were statistically analyzed using one-way ANOVA (see Table 2) to determine whether DIDI significantly outperforms the RAW method. The positive values of $\Delta Acc$ indicate that DIDI achieved higher accuracy than RAW across all tasks. Statistical significance was confirmed for all comparisons except the T1-T2 task, where the observed accuracy difference is the smallest ( $\Delta Acc = 0.45\%$). While achieving statistical significance for this task would require an increased number of experimental repetitions, we believe the observed difference is too small to offer practical value. Therefore, further repetitions were not pursued.

**Table 2:** One-way ANOVA comparing RAW and DIDI methods

task	$\Delta Acc$ [%]	p
T0-T1-T2	1.41	**3e-6**
T0-T1T2	2.13	**1e-13**
T1-T2	0.45	0.1390
emotions	9.41	**5e-22**

$\Delta Acc$ denotes the accuracy difference (positive values indicate higher accuracy for DIDI). Significant p-values, confirming a meaningful difference between the methods, are highlighted in bold

**Fig. 6:**
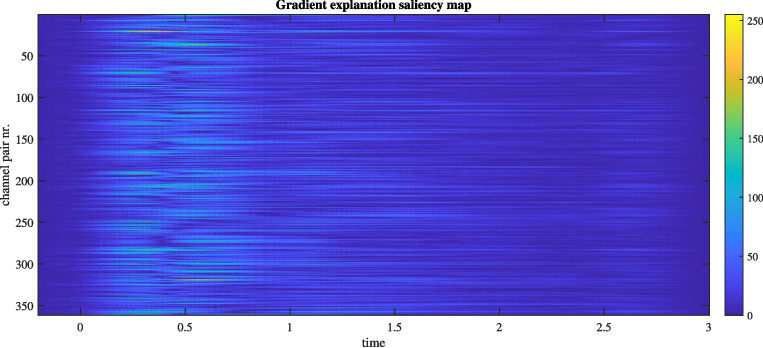
Gradient-based saliency map for a single epoch of event type T1 using the DIDI method for classification task T0-T1T2. The map illustrates the importance of each of the $M \times M$ channel pairs over time for the classification decision. When the model is successfully trained, it highlights the connections and processes that differentiate the identified class from other classes. For a clearer representation, this information can be visualized using connectivity support maps (see Fig. 7)

**Fig. 7:**
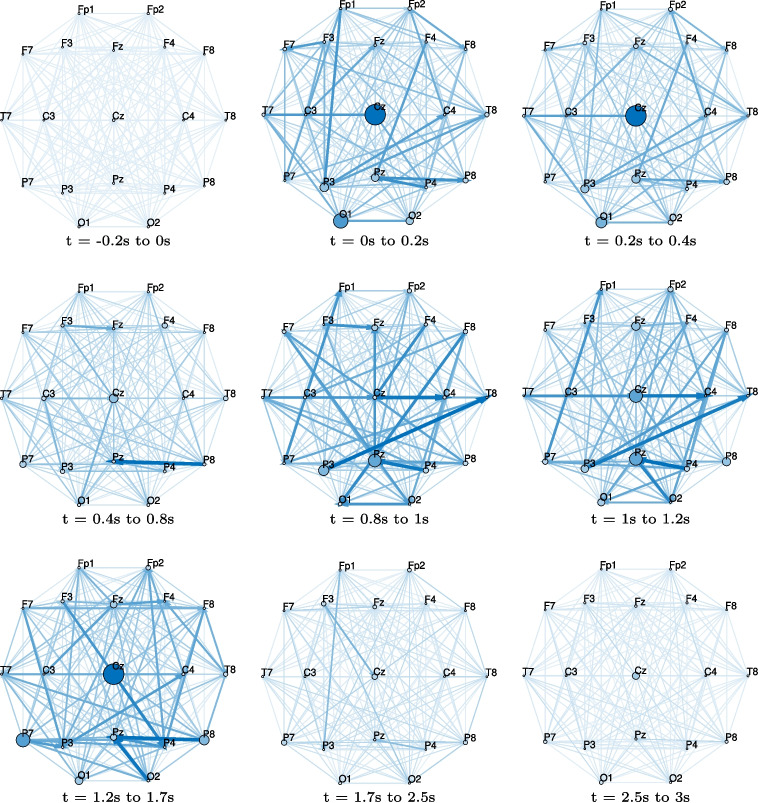
Connectivity support maps for a single epoch from the testing subset corresponding to the T1 event. These maps illustrate brain connections and processes that distinguish the identified class from others across different time intervals relative to the trigger event. Arrow widths represent the significance of connections, while electrode circle sizes indicate the importance of processes at specific electrode regions. This specific case (task T0-T1T2, event T1) highlights the differentiation between imagined left or right fist movement (T1 or T2) and the resting state (T0)

The generally improved classification accuracy suggests high reliability in the classification model’s reasoning, as revealed by saliency maps. While saliency maps for the RAW method highlight the significance of individual electrode signals over time, DIDI further emphasizes the importance of inter-electrode influences. This enhancement allows for a more comprehensive analysis, providing insights into both electrode-specific processes and neural connectivity. DIDI inputs include innovation and influence signals and excludes static information (regression model parameters), which makes it particularly well-suited for explaining dynamic processes. We demonstrate this for task T0-T1T2, which exhibits the most pronounced dynamic connectivity differences between the underlying brain processes. A gradient-based saliency map was computed for a randomly selected single epoch from the testing subset corresponding to the T1 event. For this analysis, the best-performing model from all 20 training runs, achieving a validation accuracy of 78.02%, was used. The computed gradient saliency map, shown in Fig. 6, highlights brain connections and processes that differentiate the current event of class T1T2 (left or right imagined movement) from those of the other class T0 (resting). For each time point, a connectivity support matrix can be obtained as a corresponding column of the saliency map, reordered back into a square matrix. For better interpretability, connectivity matrices can be visualized as connectivity support maps. As they form a temporal sequence, they can be shown in a video. A dynamic connectivity support map video for this specific case is provided as supplementary material. A more condensed representation is shown in Fig. 7, where connectivity information is averaged over key time intervals based on prominent changes detected in the saliency map (Fig. 6). Before the event (t = -0.2 s to 0 s), neither connections nor electrode processes significantly influence the classification decision. However, immediately after the event, several connections and electrodes become prominent. These influencing connections and electrodes change rapidly over time until the first phase of stabilization (t = 0.4 s to 0.8 s), marked by the dominance of the P8-Pz connection and electrode Cz. Beyond this period (t > 0.8 s), a new sequence of changes emerges, with a gradual decline in the decisive activity, with the most prominent connections becoming F3-Cz, P4-Pz and Fp1-P8. Such detailed temporal information offers valuable insights for neuroscientific research by facilitating the identification and differentiation of brain processes associated with distinct activities.

**Fig. 8:**
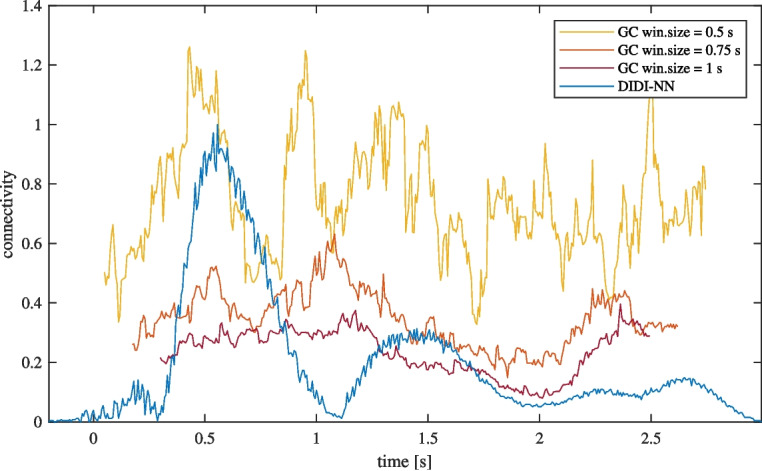
Comparison of connectivity estimates obtained using the proposed method and windowed GC with window sizes of 0.5 s, 0.75 s and 1 s. Smaller window sizes increase volatility, which may be interpreted as noise, while larger windows reduce temporal precision and sensitivity. Unlike GC, which lacks a task-specific focus, the proposed method (DIDI-NN) provides task-related connectivity estimates for each time sample without windowing. The signals involved in this analysis are shown in Fig. 1

To highlight the differences between our proposed method and conventional dynamic connectivity estimation, we compare connectivity estimates for a single electrode pair. We focus on the pair with the highest connectivity variance, $P8 \rightarrow Pz$, which also emerges as one of the most prominent. We compare connectivity estimates obtained using our method with those derived from windowed Granger causality (GC) with three different window sizes: 0.5 s, 0.75 s, and 1 s (see Fig. 8). Note that our method estimates task-related connectivity at each time sample, while windowed GC provides connectivity estimates over window-sized time intervals without focusing on specific tasks. Narrow GC windows produce highly volatile and potentially noisy estimates. Larger windows improve stability but reduce temporal precision and sensitivity. In contrast, our DIDI-NN method captures connectivity dynamics clearly while maintaining low noise.

### Static Analysis for Emotion Classification

Static analysis is demonstrated on the classification of emotions using the SEED dataset (Zheng and Lu, 2015; Duan et al., 2013). Positive, neutral or negative emotions were induced by showing 15 carefully selected movie clips each lasting 4 minutes. In total, the dataset includes 675 EEG segments, each exceeding 3 minutes in duration and equipped with the ground truth of the corresponding emotion class (positive 1, neutral 0 and negative -1). Data have been preprocessed and downsampled to 200Hz by the authors of the dataset. The recordings were conducted using a 62 channel system, however, we used only 19 channels corresponding to EEG electrodes of the international 10-20 EEG electrode placement system. Also, we decided to classify segments based on only 4s sections randomly cut out from the second minute of the provided segments. The dataset was randomly split into training and testing subsets, such that 70% of all segments were used for training and the other 30% were used for testing. The data split was based on the recording sessions, such that recordings for all 15 video clips in a session were always in the same subset. The additional validation subset was not needed as the neural network’s meta-parameters were set according to EEGNet recommendations and were not being tuned. This was so because our priority was a fair comparison of methods rather than the highest possible classification accuracy. The same data split was used for both, RAW and DIDI method. The order for the regression models was 20, which corresponds to a 100 *ms* time window of historical signal values. For each method, we ran the experiment 20 times to reduce the influence of random initialization of the neural network learnable parameters and enable statistical comparison of methods’ classification accuracy. The dataset was balanced, with a baseline (zero-rate) accuracy of 33%. During training, for both methods, we observed a significant difference between training and validation accuracies, suggesting potential model overfitting. While the training loss decreased continuously, the loss on the test set stabilized early. Interestingly, the relative performance trends were consistent between the train and test sets; however, the final training set accuracies were up to 40% higher than those measured on the test set. The test set accuracies are compared in Fig. 5 (d). To assess the statistical significance of these results, we analyzed them with one-way ANOVA, and the results are included in Table 2. DIDI method was shown to provide significantly higher accuracies than RAW.

**Fig. 9:**
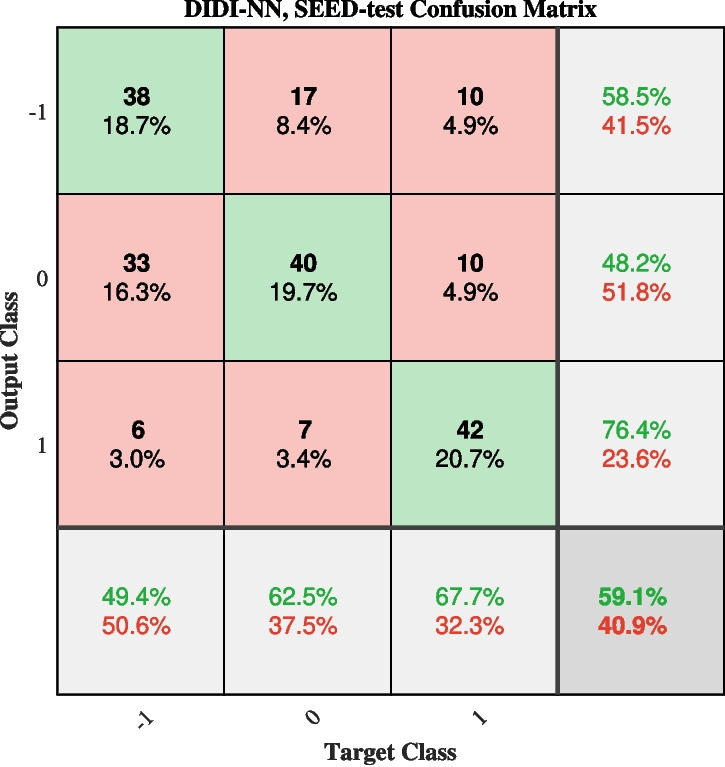
Confusion matrix for classification of emotions: negative (-1), neutral (0) and positive (1), for the best performing run of DIDI-NN method

**Fig. 10:**
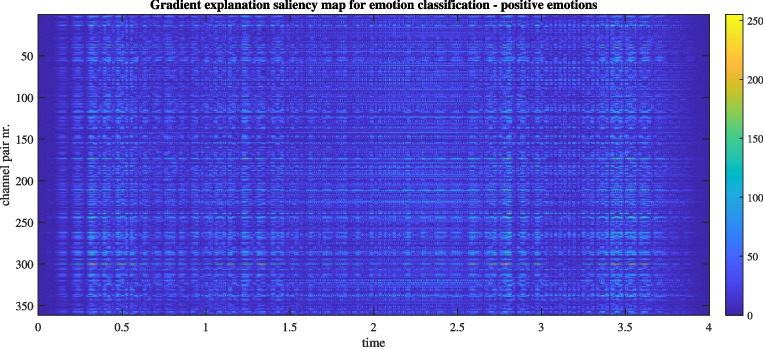
An example of a gradinet explanation saliency map for watching a video clip inducing positive emotions. For a recording from training data, generated using the best performing run of DIDI-NN method

**Fig. 11:**
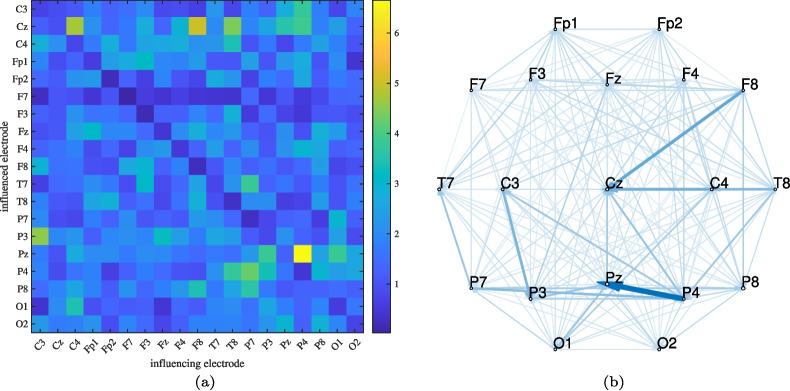
Static decision support matrix (a) and static connectivity support map (b) showing brain connections that differentiate positive emotions from negative and neutral ones. Both are generated by temporal averaging the saliency map from Fig. 10. Arrow widths indicate the importance of connections while sizes of electrode circles indicate the importance of processes confined to individual electrodes

Let us focus again on the task-related connectivity analysis, for which we selected the best-performing model among all 20 training runs, with the test set accuracy of 59.1%, and the training accuracy of 84.9%. The confusion matrix is shown in Fig. 9. It reveals that positive emotions are more easily distinguished from negative and neutral ones, while the latter two are more challenging to differentiate. This observation motivated further analysis to understand how positive emotions differ from negative and neutral ones. To explore this, a random segment representing a positive emotion was selected, and a 4-second section was randomly extracted. This segment was correctly classified, and its corresponding saliency map is displayed in Fig. 10. In the absence of a temporal reference or saliency map dynamics, we conducted a static analysis. Specifically, the saliency map was averaged along the temporal dimension and reshaped to produce a static connectivity support matrix, as shown in Fig. 11, left. The same information is also presented as a static connectivity support map in Fig. 11, right. The map highlights several influential connections, with a few standing out as particularly important, including P4-Pz, F8-Cz, C4-Cz, and C3-P3.

## Discussion

The key advantage of the proposed DIDI-NN method is its ability to obtain connectivity patterns associated with specific brain processes through the explainability of classification decisions. The resulting connectivity support maps provide detailed connectivity information with a temporal resolution matching the data sampling rate. This level of precision is unattainable with conventional connectivity estimation methods, which rely on temporal windowing that reduces their temporal resolution. In literature, the dynamic connectivity analysis rarely uses window sizes below one second and only in extreme cases, the obtained temporal resolution can be as low as 50 *ms* (Šverko et al., 2024), which is still high above the EEG data sampling period achieved by our proposed approach. In addition, the obtained connectivity reflects the differences between analyzed cognitive processes, thus determining which specific connections are associated with them, also unreachable by conventional methods.

The trustworthiness of the obtained explanations is directly linked to the classification accuracy. Consequently, we first analyzed how the proposed dynamic influence data inflation (DIDI) method influences classification performance. To ensure unbiased comparisons, we used the same classifier for all tests, i.e., EEGNet (Lawhern et al., 2018) that was not tailored specifically for our proposed method. Classifier optimization was deliberately avoided, as it could introduce additional biases. Instead, the meta-parameters recommended by EEGNet’s authors were used. Consistency was further ensured by applying the same random split of data into train and test subsets for both methods, repetitions, and tasks (except for the emotion classification due to using a different dataset). To address uncertainties arising from the random initialization of neural network weights during training, we repeated the training and testing process 20 times for each method and task, and statistically analyzed the results using ANOVA.

Given our focus on ensuring a fair comparison, the achieved classification performance is not optimal. Higher accuracies could be achieved by optimizing the meta-parameters of the classification model or employing alternative neural network architectures. Notably, any end-to-end neural network classification architecture proposed for EEG could be utilized.

It’s important to note that innovation signals, given a known regression model, can perfectly reconstruct the raw signals. This suggests that the innovation signals retain all the dynamic information related to brain activity, while static information is embedded in the autoregression models. These models can be interpreted as directed transfer functions that define the system’s spectral properties (Dissanayaka et al., 2015; Geng et al., 2020). Interestingly, despite the apparent reduction of information kept by DIDI, classification results are generally improved. This may be unexpected particularly for the emotion classification task, as previous studies have found correlations between emotions and power within specific frequency bands (Wang and Wang, 2021; Adhikari et al., 2025). However, it is expected that the proposed method enhances classification performance when the targeted brain processes are characterized by distinct connectivity patterns. This makes the approach valuable not only for improving explainability, but also for boosting classification accuracy in relevant tasks.

The absence of static information in innovation signals, without predictable (autoregressive) components, makes them less susceptible to static influences, e.g., subject state or baseline activity. While this may pose challenges for tasks that rely on static information, such as emotion classification, it may simultaneously enhance sensitivity to transient, dynamic interactions. When GC is applied to innovation signals rather than raw EEG data, the resulting connectivity estimates are generally consistent with those from raw data. However, the removal of static components can lead to improved robustness in classifying processes that rely on dynamic neural activity.

The proposed DIDI-NN method enables classification decisions to be explained in terms of dynamic directed connectivity and process dynamics for each individual EEG segment being classified. It is crucial to emphasize that these results indicate which connections differentiate a given class from others, rather than simply showing which connections are active for a specific class. This distinction is fundamental to correctly interpreting the findings. Consequently, direct comparisons between connectivity patterns obtained via DIDI-NN and those estimated by conventional GC method are not straightforward. Conventional approaches to connectivity analysis rely on statistical testing over the entire dataset (e.g., p-values) to identify significant connections, whereas DIDI-NN directly delivers time-resolved, context-sensitive explanations at the level of individual epochs. Given the high degree of inter-subject variability in EEG data, conventional statistical methods may overlook relevant individual-level patterns. Nevertheless, it is reasonable to expect that connections consistently identified as influential in DIDI-NN classifications would show correspondence with GC-derived connections that are statistically significant at the population level.

The connectivity information obtained by DIDI-NN is inherently dynamic, but it can be averaged over time to derive static connectivity patterns. Since visualizing connectivity support maps for all individual samples is often impractical, except as frames of video sequences, time-averaged maps can be more convenient, as demonstrated for motor movement analysis in Fig. 7. This approach can be extended to analyze multiple EEG segments. If a clear temporal reference is available, such as the time of stimulation events, the classification decision logic can be analyzed statistically. The simplest form of such an analysis involves averaging connectivity patterns over multiple epochs. More advanced statistical approaches, however, open up further opportunities for deeper insights. These may include estimating variability across subjects or identifying significant differences between subject groups.

As previously discussed, the reliability of the obtained explanations is directly tied to the classification accuracy, which must be evaluated on data not used for training to avoid bias. However, this condition does not necessarily apply to the generation of classification explanations. Trained models inherently contain knowledge derived from the training data, and this knowledge influences both the model’s classification capabilities and explainability. Importantly, explanation decisions are not compromised even if obtained from the training data, while their reliability still depends on the true classification accuracy. This perspective aligns with the use of GC, which computes the regression models from the same signal for which it later estimates the univariate and bivariate prediction errors and connectivity. Therefore, explanations generated from training data in this context are still valid, as the extraction of meaningful insights relies on the integrity of the underlying model. In our experiments, an epoch from the testing set was used for analysis in the imagined motor imagery experiment and a recording section from the training set for the emotion classification experiment.

Our overall classification accuracy is lower than reported in some other studies (for emotion classification see Prabowo et al. (2023)). This is not only due to the use of an unoptimized model but also due to the more challenging analysis conditions. Specifically, emotions were classified from 4-second EEG segments, even though recordings of nearly 4 minutes were available. Additionally, only 19 electrodes following the international 10-20 system were employed, despite the availability of a larger number of electrodes. These constraints were imposed to address computational complexity, which represents the primary drawback of using the proposed dynamic influence data inflation method for training neural network classifiers. Training end-to-end neural network classifiers is inherently more computationally demanding than conventional classification models, and this limits the length of input EEG segments that can be processed. Furthermore, dynamic influence data inflation significantly increases computational requirements by squaring the number of channels, as a separate channel is created for each electrode pair.

Some additional caution is needed when interpreting the results. The inflated influence data used for classification consists of both, influence signals and innovation signals. As influence signals are extracted from innovation signals, information provided to the classifier is duplicated. As a consequence, the classifier may still rely on the innovation signals, even when the same information is contained in influence signals. This could potentially reduce the quality of the neurological insights extracted. However, since the connectivity is readily available in the added signals and only indirectly in the original ones, it can be expected that they will be prioritized by the neural network during training whenever they are relevant. This is because the backpropagation algorithm used for neural network training adapts the model to focus on the most informative features.

The above mentioned data redundancy will be addressed in future work. One potential solution is to replace the innovation signals with stripped versions that exclude their influence information. Other challenges for future work arise from employing the method for analysis in practice. This would require optimization of the neural network classifier and testing the compliance of results with existing neurological understanding of brain function.

Another potential direction for future work involves performing connectivity analysis at the source level, which is often proposed to address limitations related to volume conduction and to potentially enhance the anatomical interpretability of connectivity patterns. Although volume conduction can affect some connectivity metrics, it has a limited influence on regression-based approaches such as Granger causality (GC), which underlies our method. Because volume-conducted components are shared instantaneously across electrodes, they are already captured by univariate regression and do not further reduce the prediction error in bivariate models, making them unlikely to produce spurious directed connections. Nevertheless, source-level analysis may offer complementary benefits. In particular, it could reduce computational complexity by lowering the dimensionality of the data and may yield more interpretable results when sources correspond to known functional regions. However, source localization introduces additional assumptions and may also lead to misleading interpretations (Pester and Ligges, 2018).

## Conclusion

In this work, we proposed an approach for estimating task-related dynamic brain connectivity, which is contrasting the conventional signal classification approaches. Instead of reducing data through feature extraction, our method inflates the data by adding components that are meaningful for explaining classification decisions. This not only improves the interpretability of the classification decisions but also enhances classification accuracy.

We introduced the dynamic influence data inflation technique (DIDI) for extracting signals related to connectivity, based on the well-established Granger causality (GC) connectivity estimation method.

By employing gradient-based saliency maps, we can analyze classification decisions, providing insights into the importance of specific functional brain connections at each time point and for each epoch. These connections can be visualized through connectivity support maps, which are intuitive and facilitate the interpretation of underlying neurological processes. The dynamic connectivity information can also be presented as video sequences, offering an engaging and clear format for presenting these insights.

Two main advantages distinguish our approach from standard connectivity estimation methods. First, it provides high temporal resolution, revealing connectivity for each individual sample for each individual input sequence. Second, it allows us to identify the specific connections associated with particular cognitive processes, isolating them from other brain activity.

This study lays the groundwork for improved connectivity analysis thereby contributing to more robust neuroscientific research. Future research will build upon this for in-depth analysis of specific cognitive processes with optimized neural network architectures.

Finally, data inflation for explainability is not limited to connectivity. Other neurologically meaningful signal components, such as frequency bands, can also be extracted and integrated similarly, offering further opportunities to enhance the interpretation of brain function.

## Supplementary Information

Provided is a supplementary video of a dynamic connectivity support map for the imaginary movement task generated from a single randomly selected epoch corresponding to the T1 event (imaginary movement).

## Information Sharing Statement

The EEG datasets used in this study are publicly available:

- Motor Movement/Imagery Dataset: PhysioNet (https://physionet.org/content/eegmmidb/1.0.0/)
- SEED Emotion Dataset: (https://bcmi.sjtu.edu.cn/~seed/seed.html)

The code implementing the Dynamic Influence Data Inflation with Neural Network explainability (DIDI-NN) method, along with scripts for training, evaluation, and visualization of connectivity results, is available at: https://doi.org/10.5281/zenodo.14692736

## Supplementary Information

Below is the link to the electronic supplementary material.

Supplementary file 1 (avi 57633 KB)

## Data Availability

The datasets used in this study were obtained from publicly available sources, as referenced.

## Code Availability

The code for conducting experiments from this paper is openly available, see: 10.5281/zenodo.14692736.
